# Predicting survival after pulmonary metastasectomy for colorectal cancer: previous liver metastases matter

**DOI:** 10.1186/1471-2482-10-17

**Published:** 2010-06-03

**Authors:** Ulrich Landes, John Robert, Thomas Perneger, Gilles Mentha, Vincent Ott, Philippe Morel, Pascal Gervaz

**Affiliations:** 1 Department of Surgery, Geneva University Hospital and Medical School, Geneva, Switzerland; 2 Department of Biostatistics, Geneva University Hospital and Medical School, Geneva, Switzerland

## Abstract

**Background:**

Few patients with lung metastases from colorectal cancer (CRC) are candidates for surgical therapy with a curative intent, and it is currently impossible to identify those who may benefit the most from thoracotomy. The aim of this study was to determine the impact of various parameters on survival after pulmonary metastasectomy for CRC.

**Methods:**

We performed a retrospective analysis of 40 consecutive patients (median age 63.5 [range 33-82] years) who underwent resection of pulmonary metastases from CRC in our institution from 1996 to 2009.

**Results:**

Median follow-up was 33 (range 4-139) months. Twenty-four (60%) patients did not have previous liver metastases before undergoing lung surgery. Median disease-free interval between primary colorectal tumor and development of lung metastases was 32.5 months. 3- and 5-year overall survival after thoracotomy was 70.1% and 43.4%, respectively. In multivariate analysis, the following parameters were correlated with tumor recurrence after thoracotomy; a history of previous liver metastases (HR = 3.8, 95%CI 1.4-9.8); and lung surgery other than wedge resection (HR = 3.0, 95%CI 1.1-7.8). Prior resection of liver metastases was also correlated with an increased risk of death (HR = 5.1, 95% CI 1.1-24.8, p = 0.04). Median survival after thoracotomy was 87 (range 34-139) months in the group of patients without liver metastases versus 40 (range 28-51) months in patients who had undergone prior hepatectomy (p = 0.09).

**Conclusion:**

The main parameter associated with poor outcome after lung resection of CRC metastases is a history of liver metastases.

## Background

Resection of hepatic metastases from colorectal cancer (CRC) has yielded 5-year survival rates ranging from 25% to 50% [1,2]. Similarly, resection of lung metastases from CRC has yielded 5-year survival rates ranging from 20% up to 60% in large series [3,4]. Based on these encouraging results, many surgeons have expanded the indications for resecting metastatic CRC, and there is nowadays growing pressure to perform lung metastasectomy, even in asymptomatic CRC patients. The issue therefore is to select the patients with pulmonary metastases who are good candidate for surgical therapy with a curative intent. Unfortunately, it is currently not possible to do so - hence the necessity for surgeons to preoperatively identify clinico-pathological parameters predicting survival after thoracotomy.

LM develop in 5-15% of CRC patients according to two different scenarios: the first scenario, most common, is the metachronous development of lung metastases in a patient who has previously developed in transit liver metastases; in the second scenario - less frequent - patients develop lung metastases synchronous or metachronous to primary colorectal cancer, but without evidence of liver metastases ("skip metastases") [5]. In the latter situation, the reason why the liver does not provide an adequate soil for the metastases to develop is unclear, but might involve, among other factors, deficient tumor angiogenesis [6]. Most CRC patients included in surgical series of pulmonary metastasectomy belonged to the second category.

Since 2000, about 20 series have investigated the outcome of CRC patients who underwent resection of lung metastases with a curative intent. Reported 5-year overall survival rates range from 24% [7] to 68% [8], indicating that these studies reflect the experience of highly specialized centres with a selected subset of patients [9]. Various factors associated with prolonged survival after surgery for lung metastases from CRC have been identified, including: a) a long disease-free interval (defined as the time from colectomy to the development of lung metastases [10-12]; b) prethoracotomy carcinoembryogenic antigen (CEA) level <5NG/ML [13-15]; c) a single isolated metastasis < 3 cm in size [16-18]; and d) the absence of thoracic lymph node invasion [19,20]. By contrast, a history of previous liver metastases has never been documented as statistically significant. However, many series of pulmonary metastasectomy for CRC included mostly patients without liver metastases, or were conducted at a time when hepatectomy was rarely considered in patients with extensive liver metastases. The present study was conducted in order to assess the impact of prior surgery for liver metastases on the outcome of CRC patients who subsequently underwent pulmonary metastasectomy.

## Methods

We performed a retrospective analysis of all CRC patients who underwent thoracotomy for lung metastases with a curative intent in our institution since 1996. Lung surgery was performed in the Thoracic Surgery Unit and both resections of primary tumor and liver metastases were performed in the Visceral Surgery Unit of Geneva University Hospital. We included all patients with a histopathological diagnosis of colorectal adenocarcinoma metastatic to the lung, whether or not they underwent prior liver surgery. The following parameters were recorded and considered for statistical analysis: 1) patients' characteristics; age, gender: 2) primary tumor characteristics; TNM stage; location (colon or rectum); preoperative radiation therapy; adjuvant chemo-and/or chemotherapy: 3) characteristics of liver metastases when present; number of metastases and location (uni- versus bilobar); size of largest metastases: adjuvant chemotherapy; and type of surgery (metastasectomy versus segmentectomy versus right/left hepatectomy; 4) characteristics of lung metastases: number and size of the largest metastases; type of surgery performed (wedge versus lobectomy versus pneumonectomy); presence of involved mediastinal lymph nodes; adjuvant chemotherapy; disease-free interval between primary tumor resection and development of liver/lung metastases; and location of lung metastases (uni-versus bilateral). The study was conducted in accordance with institutional guidelines of the Ethics Committee for Clinical Research of Geneva University Hospital.

Eligibility for surgical resection of lung metastases from CRC was based upon four criteria: 1) control of primary tumour considered as achieved; 2) absence of extrathoracic lesions at the time of lung surgery; 3) possibility to perform a complete resection (R0) of pulmonary metastases; and 4) adequate pulmonary reserve to tolerate the planned resection, in accordance with classic oncologic criteria for pulmonary metastasectomy. Follow-up was performed in the Surgical Oncology Unit, with repeated clinical examination and thoracoabdominal CT scan imaging once a year for 5 years to detect local or systemic tumor recurrence. In addition all patients underwent colonoscopy surveillance at 1, 3 and 5 years post colectomy.

### Statistical analysis

Survival was assessed from the time of thoracotomy to the time of last follow-up, and statistical analysis was performed using SPSS^® ^software version 17.0 (SPSS, Chicago, Illinois, USA). Continuous variables were collapsed into clinically meaningful categories and compared using chi-square^2 ^tests. Logistic regression was used to identify variables associated with either tumor recurrence or death. We considered for the multivariate model all variables with univariate p-values < 0.20, and retained in the final model all variables with an adjusted p-value < 0.1. Survival curves were calculated according to the Kaplan-Meier method and difference between groups were analyzed with log-rank test. All tests were two-sided and a p value < .05 was considered statistically significant.

## Results

Between February 1996 and July 2009, forty patients (median age 63.5 [range 33-82] years) underwent resection of pulmonary metastases from CRC. Median follow-up was 33 (range 4-139) months. Twenty-four (60%) patients did not have previous liver metastases before undergoing lung surgery. Sixteen patients underwent resection of liver metastases with a curative intent. Median disease-free interval between liver surgery and the development of lung metastases was 17 (range 0-60) months. Median disease-free interval between primary colorectal tumor and development of lung metastases was 32.5 (range 0-82) months. 27 (67%) patients presented with a single lung metastasis, and 24 (60%) of lung metastases were < 3 cm in size. Prethoracotomy CEA serum level was missing in 22 patients and was elevated in 11 of the remaining 18 patients. This parameter was therefore not considered for inclusion in statistical analysis. The clinical and pathological characteristics of these 40 patients and their tumors are summarized in Table 1.

**Table 1:** Clinical and Pathological characteristics of 40 crc patients who underwent lung metastasectomy

**Gender **(F/M)	13/27
**Age **(median, range) years	63.5 (33-82)
Primary Tumor T stage	
1	3
2	14
3	16
4	7
**Liver metastases**	
Yes/No	16/24
**Liver metastases Number **(median, range)	2 (1-8)
**Liver metastases Location**	
Unilobar (right/left)	9/4
Multilobar	3
**Lung metastases Number**	
Single	27
Multiple	13
**Lung metastases Size**	
< 3 cm	24
> 3 cm	16
**Type of lung resection**	
Wedge resection or segmentectomy	32
Lobectomy	7
Pneumonectomy	1
**Disease-free interval **median (range) months	32.5 (0-82)
**Colectomy to lung metastases**	
**Disease-free interval **median (range) months	17 (0-60)
**Hepatectomy to lung metastases**	

**Figure 1:**
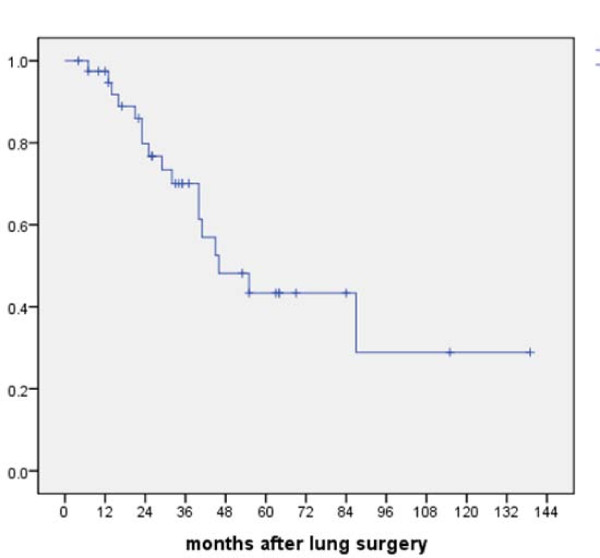
**Kaplan-meier overall survival of 40 colorectal cancer patients who underwent resection of lung metastases**.

**Figure 2:**
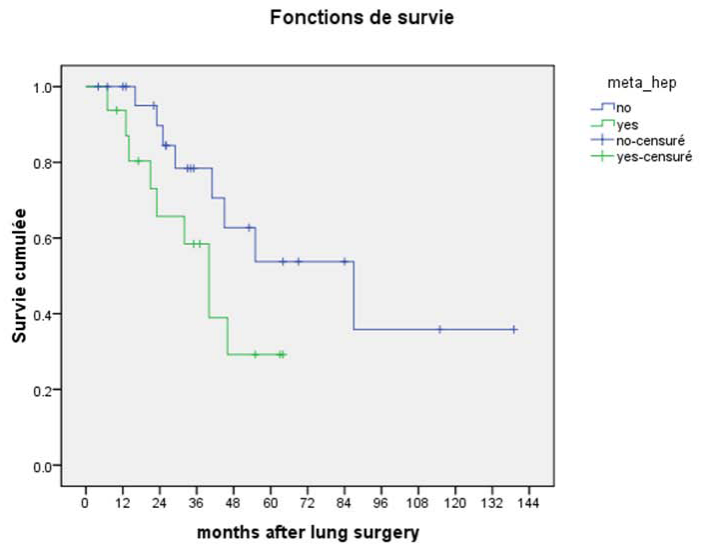
**Kaplan-meier overall survival of colorectal cancer patients who underwent resection of lung metastases according to the presence or not of prior liver metastases**.

There were no postoperative deaths. For the whole group, 3- and 5-year overall survival rates after thoracotomy were 70.1 and 43.4%, respectively (Figure 1). In univariate analysis, the presence of synchronous liver and/or lung metastases in the primary tumor (M1/stage IV) was associated with a high risk for tumor recurrence after thoracotomy (HR = 3.1; 95% Confidence Interval 1.2-7.8, p = 0.01) (Table 2). In multivariate analysis, two parameters were correlated with tumor recurrence after thoracotomy; 1) a history of previously resected liver metastases (HR = 3.8, 95%CI 1.4-9.8); and 2) lung surgery other than wedge resection (HR = 3.0, 95%CI 1.1-7.8). Prior surgery for liver metastases was also correlated with an increased risk of death (HR = 5.1, 95% CI 1.1-24.8, p = 0.04) (Table 3). Median survival after thoracotomy was 87 (range 34-139) months in the group of patients without liver surgery versus 40 (range 28-51) months in patients who underwent prior hepatectomy for liver metastases (log-rank test, p = 0.09). 5-year overall survival rate was 53.8% in the group of patients with isolated lung metastases versus 29.2% in the group of patients who had undergone prior liver metastasectomy (Figure 2).

**Table 2:** Relative hazards of recurrence or death in patients with pulmonary metastases secondary to colo-rectal cancer (univariate analysis in cox proportional hazards model, time since operation for lung metastasis)

	Recurrence		Death	
***Gender ***(F versus M)	2.1 (0.8 - 5.1)	0.12	1.7 (0.6 - 4.4)	0.30
***Age ***(per 10 years)	0.84 (0.52 - 1.34)	0.46	1.05 (0.61 - 1.79)	0.86
***Primary Tumor T stage***		0.34		0.60
1-2	1.0		1.0	
3	4.0 (0.5 - 30.5)		2.8 (0.4 - 21.7)	
4	2.6 (0.3 - 23.3)		3.0 (0.3 - 27.1)	
***Tumor 3-4 (vs. 1-2)***	3.6 (0.5 - 26.8)	0.22	2.8 (0.4 - 21.6)	0.32
***Primary Tumor N stage***		0.23		0.42
0	1.0		1.0	
1	2.3 (0.8 - 6.9)		1.7 (0.5 - 5.6)	
2	2.7 (0.8 - 9.3)		2.2 (0.6- 7.8)	
***Node 1-2 (versus 0)***	2.4 (0.9 - 6.7)	0.09	1.9 (0.7 - 5.5)	0.22
***Primary tumors M stage***	3.2 (1.1 - 8.9)	0.03	1.4 (0.5 - 4.5)	0.53
M1 (versus M0)				
***Stage 3-4 (versus 1-2)***	2.4 (0.9 - 6.7)	0.09	1.8 (0.6 - 5.1)	0.28
***Liver metastasis***	3.1 (1.2 - 7.8)	0.01	2.3 (0.8 - 6.2)	0.10
***Time primary to lung ***(per year)	0.9 (0.6 - 1.2)	0.51	1.0 (0.8 - 1.4)	0.87
***Time primary to first metastasis ***(per year)	0.71 (0.49 - 1.02)	0.06	0.9 (0.6 - 1.2)	0.45
***Multiple versus single lung metastasis***	1.2 (0.5 - 3.3)	0.67	1.2 (0.9 - 1.7)	0.17
***Size lung metastasis >20 mm***	1.8 (0.7 - 4.8)	0.21	1.2 (0.4 - 3.2)	0.76
***Neo-adjuvant treatment for lung metastasis***	2.8 (0.6 - 13.0)	0.20	3.4 (0.9 - 12.4)	0.06
***Adjuvant treatment for lung metastasis***	0.5 (0.1 - 3.9)	0.52	1.1 (0.3 - 4.9)	0.89
***Lung operation other than wedge ***(versus wedge)	2.3 (0.9 - 5.9)	0.06	1.8 (0.7 - 5.0)	0.23

**Table 3:** Multivariate analysis of risk factors for tumor recurrence and death

	**Recurrence**		**Death**	
		
***Node 1-2 ***(versus 0)	1.7 (0.6 - 5.0)	0.34	1.5 (0.5 - 4.8)	0.48
***Time primary to first metastasis ***(per year)	0.83 (0.48 - 1.44)	0.51	1.3 (0.8 - 2.1)	0.35
***Liver metastasis***	2.7 (0.7 - 10.0)	0.14	5.1 (1.1 - 24.8)	0.04
***Size lung metastasis >20 mm***	2.1 (0.6 - 6.6)	0.23	0.9 (0.3 - 2.7)	0.86
***Multiple versus single lung metastasis***	1.4 (0.4 - 4.6)	0.58	3.4 (0.9 - 12.9)	0.07

## Discussion

Our series differs from most published reports focusing on pulmonary metastasectomy, because of the high (40%) percentage of patients who underwent surgery for liver metastases prior to thoracotomy. With a median follow-up of 33.5 and 64 months after thoracotomy and colectomy, respectively, 17 (42.5%) patients are alive and disease-free, with an overall 5-year survival rate of 43%. The main parameter associated with both tumor recurrence (HR = 3.8) and death (HR = 5.1) after thoracotomy is a history of resected liver metastases. Median survival after pulmonary metastasectomy was twice longer in CRC patients without previous liver surgery (87 versus 40 months).

With recent advances in surgical technique as well as adjuvant chemotherapy, many CRC patients treated with a curative intent for liver metastases have a prolonged survival; in specialized centres (and in selected patients), 5-year overall survival rate after hepatectomy for CRC liver metastases exceeds 50% [21]. Unfortunately, some of them will eventually develop extra-hepatic tumor recurrence, usually in the lung and may be candidates to additional pulmonary metastasectomy; most clinicians would concur that R0 resection of pulmonary metastases may improve survival in this subset of patients [22]. Whether these patients have identical survival benefit in comparison with those patients who developed isolated lung metastases remains controversial [23]; the data presented herein, however, clearly indicates that prior surgery for liver metastases is associated with a higher risk for tumor recurrence and death after pulmonary metastasectomy. These results appear hardly surprising (patients with both liver and lung metastases are more likely to have systemic disease), yet the correlation between prior liver metastasectomy and poor survival in patients undergoing pulmonary metastasectomy had not been reported so far.

Riquet et al. reported a series of 127 patients who underwent pulmonary metastasectomy; twenty-nine (23%) of them had undergone previous liver surgery for liver metastases. Overall 5-year survival was similar to ours (41%), but there was a trend towards *better *survival in patients who had undergone previous resection of liver metastases [24]. In addition, five recent series (800 patients in total) have failed to identify prior liver metastases as a risk factor for poor outcome after lung metastasectomy in CRC patients [8,16,19,20,25]. In the largest series of pulmonary metastasectomy for CRC, the presence of extrathoracic disease was an independent predictive factor for poor survival, but it was unclear whether these patients had prior liver surgery or not [26].

Our study has several limitations, mostly related to the small number of patients and the fact that perioperative management of patients was not uniform in terms of (neo) adjuvant chemotherapy. There is certainly a possibility for introducing selecting bias in non-randomized studies. The strength of our series is related to the high percentage of patients who underwent prior liver surgery, allowing for sound comparison of survival between groups with adequate statistical power despite limited number of events. Finally, this series reflects the outcome of a relatively homogenous population of patients who were treated according to similar clinical guidelines, by a small group of surgeons over a relatively short period of time.

## Conclusion

A minority of CRC patients (10%) with lung metastases are candidates for surgery with curative intent. In this selected category of patients, a subset has previously developed liver metastases, which were resected with a curative intent. The question of whether to perform pulmonary metastasectomy in this situation will get more complex with advances in chemotherapy. A history of previous liver metastases is certainly not a contra-indication for pulmonary metastasectomy, but the data presented here indicates that these patients have a higher risk of tumor recurrence and a decreased survival in comparison with patients who underwent surgery for lung-only CRC metastases. These results are clinically relevant, since the incidence of isolated lung metastases without associated liver metastases is low (<10%); a history of previously resected liver metastases should be considered a poor prognostic factor in the small subset of CRC patients candidates for pulmonary metastasectomy.

## Competing interests

The authors declare that they have no competing interests.

## Authors' contributions

**UL: **protocol design, data collection. **VO: **data collection. **JR: **protocol design, editing manuscript. **GM: **editing manuscript. **TP: **statistical analysis. **PM and PG: **final review, supervision of scientific content of manuscript. All authors have read and approved the final version of manuscript

## Pre-publication history

The pre-publication history for this paper can be accessed here:

http://www.biomedcentral.com/1471-2482/10/17/prepub
